# Biogenic Ceria Nanoparticles (CeO_2_ NPs) for Effective Photocatalytic and Cytotoxic Activity

**DOI:** 10.3390/bioengineering7010026

**Published:** 2020-03-13

**Authors:** Siripireddy Balaji, Badal Kumar Mandal, L. Vinod Kumar Reddy, Dwaipayan Sen

**Affiliations:** 1 Trace Elements Speciation Research Laboratory, Department of Chemistry, School of Advanced Sciences, Vellore Institute of Technology, Vellore 632014, India; siripireddybalaji@gmail.com; 2 Cellular and Molecular Therapeutics Laboratory, Centre for Biomaterials, Cellular and Molecular Theranostics, Vellore Institute of Technology, Vellore 632014, India; vinodkumarreddy.l@vit.ac.in (L.V.K.R.); dwaipayan.sen@vit.ac.in (D.S.)

**Keywords:** *E. globulus* extract, CeO_2_ NPs, photocatalytic activity, human carcinoma cells, ROS, cytotoxic activity

## Abstract

Ceria nanoparticles (CeO_2_ NPs) are generally considered in various functional applications, such as catalysts in fuel cells, sensors, and antioxidant and oxidase-like enzymes in the biological environment. The CeO_2_ NPs were synthesized using the *E. globulus* leaf extract-mediated hydrothermal technique. The synthesized NPs were characterized by various analytical instruments including powder X-ray diffractometer (PXRD), scanning electron microscope (SEM), transmission electron microscope (TEM) and dynamic light scattering (DLS) analysis. The XRD results showed an average NPs sizes of 13.7 nm. Cytotoxic study results showed an IC_50_ value of 45.5 µg/L for A549 and 58.2 µg/L for HCT 116, indicating that CeO_2_ NPs are more toxic to A549 compared to HCT116 cell lines. The generation of ROS was responsible for its cytotoxic activity against cancer cell lines. Specific surface area (40.96 m^2^/g) and pore diameter (7.8 nm) were measured using Brunauer–Emmett–Teller (BET) nitrogen adsorption–desorption isotherms. CeO_2_ NPs with a high surface area were used as photocatalyst in degrading sunset yellow (SY) dye under UV-irradiation and 97.3% of the dye was degraded within 90 min. These results suggest that the synthesized CeO_2_ NPs could be used as a good photocatalyst as well as a cytotoxic agent against human cancer cell lines.

## 1. Introduction

Nanomaterials having a high surface area, smaller size and different shapes, allow them to be used in different applications with enhanced properties [1,2]. Ceria nanoparticles (CeO_2_ NPs) are rare earth oxide NPs that have several important inherent properties, such as oxygen storage capacity, catalysis, optics, magnetic, high thermal and chemical stability [3,4]. Currently various synthetic methods such as sol-gel, micro-emulsion, precipitation, sono-chemical, surfactant template synthesis and glycine-nitrate combustion [5,6] are adopted to synthesize NPs; however, all these methods use expensive, toxic and hazardous chemicals and solvents. Green synthesis of nanomaterials is the most favorable method because it is environmentally friendly, low-cost and non-toxic without using substances that are hazardous to the environment and mankind [7,8]. Moreover, the size of the NPs is easily controlled by varying the extract volume since phytochemicals act as a reducing as well as stabilizing agent. Green synthesis also results in a narrow size distribution of NPs. In addition, green-synthesized NPs are biocompatible due to the capping of biomolecules on the surface of the NPs. 

Normally the properties of the synthesized NPs depend on their crystallinity and phase purity, morphology, size, surface area, surface capping, band gap and stability during an activity study. Hence, a thorough characterization of the NPs is essential before designing any experimental protocol. Initially UV-Visible spectroscopy is used to check whether the formation of NPs is happening or not by comparing the absorption λ_max_ of the precursor and synthesized NPs. Then phase purity, crystallinity and grain size are determined by XRD analysis. Afterwards, a FT-IR study is carried out to check the functional groups attached due to the capping of phytochemicals on the NPs surface. Actual particle size, morphology, size distribution and lattice spacing are checked by microscopic studies using SEM, TEM and EDX. To study the photocatalytic activity, the band gap of the NPs is important and this is determined by UV-DRS spectroscopic analysis. The thermal stability of the NPs is monitored by thermogravimetric (TGA) analysis. Before the catalytic activity is investigated, the surface area is determined by Brunauer–Emmett–Teller (BET) analysis, because a good catalyst possesses a high surface area for enhanced activity. During solution-phase catalysis or adsorption studies, the stability of the NPs dispersion in the solution is highly important. For this reason dynamic light scattering (DLS) analysis is carried out to measure the zeta potential as well as the hydrodynamic diameter. Additionally, DLS helps to determine the NPs size distribution in solution. In addition, the pore volume and pore diameter are determined by DLS analysis for a better understanding of the reaction mechanism. 

CeO_2_ NPs are crystalline in nature with distinctive properties such as UV light absorbing ability, a high hardness index, high stability at high temperature and reactivity. Hence, they are widely used in various biological and photocatalytical applications [9]. CeO_2_ NPs exhibit a higher surface area and smaller particle size compared to other rare earth oxide nanoparticles. Decreasing particle size of CeO_2_ NPs enables to generate more oxygen vacancies due to large surface to volume ratio, which enables its unique properties. Moreover, CeO_2_ NPs exhibit two stable oxidation states, +4 and +3, and the relatively ease of switching between these two oxidation states is a key factor in its catalytic activity, use as a multi-enzyme type agent and other biomedical applications [10,11,12,13].

*Azadirachta indica* has been used to synthesize CeO_2_ NPs for the photodegradation of rhodamine B (RhB) dye [14]. In another study carbohydrate sugars (0.05 M) (i.e., lactose, glucose and fructose) were used to synthesize CeO_2_ NPs using the microwave method [15] and MTT [3-(4, 5-dimethylthiazol-2-yl)-2, 5-diphenyltetrazolium bromide] assay was performed to check the cytotoxicity of CeO_2_ NPs against mesenchymal stem cells after exposure to NPs (400, 200, 100, 50, 25 and 12.5 mM of 2–6 nm) for duration of 24 h, the cell viability decreased with increasing nanoparticle dose. This result suggests that CeO_2_ NPs have potential for use in biomedical applications. *Gloriosa superba L.* (*G. superba L.*) leaf extract was used to synthesize CeO_2_ NPs and its antibacterial activity was relatively more susceptible to Gram-positive bacteria compared to Gram-negative bacteria, whereas toxicological study suggested the generation of ROS [16]. Additionally, *Aloe vera arborescens* (*A. Vera arborescens*) leaf extracts were used to synthesize CeO_2_ NPs [17] and an antimicrobial study was performed against different species. The minimum inhibitory concentration (MIC) values of the studied species were 15 μg/mL *(B. subtilis*), 50 μg/mL (*S. aureus*), 20 μg/mL (*S. epidermidis*), 25 μg/mL (*E. faecalis*) and 100 μg/mL (*E. coli*), 50 μg/mL (*K. pneumoniae*) and 75 μg/mL (*P. aeruginosa*). In addition, honey, which is rich in carbohydrates, was used to prepare CeO_2_ NPs with the sol-gel technique [18] and its neurotoxicity effects on neuro2A cells showed a dose dependent toxicity above a dose of 25 mg/mL. Inflammatory response is a basic parameter that is used to study the effect of nanomaterials inside the organism [19]. 

There are a very few research articles available on the toxicological activities of CeO_2_ NPs. Although CeO_2_ NPs have multifunctional activities, they are rarely used in the photodegradation of different food coloring dyes, like sunset yellow (SY), which is a typical environmental pollutant that has little degrading (or) decomposing behavior under exposure to solar light alone.

In the present project, the main objective was to synthesize CeO_2_ NPs using an environmentally friendly non-toxic method, its characterization and photocatalytic ability to photodegradation of SY dye. In addition, we evaluated its cytotoxicity on human cancer cells such as colon carcinoma (HCT 116) and Adeno carcinoma (A549) cell lines. 

## 2. Experimental

### 2.1. Materials

Fresh *E. globulus* leaves were collected from University of Hyderabad campus, Hyderabad, India during the spring season. Cerous nitrate hexahydrate [Ce(NO_3_)_3_·6H_2_O], MTT [3-(4, 5-dimethylthiazol-2-yl)-2, 5-diphenyltetrazolium bromide], DCFH_2_-DA (2,2dichloro-diflourescein dye) and DPPH (2, 2 Diphenyl-1-picrylhydrazyl) were purchased from Sigma-Aldrich, India. Human cell lines A549 and HCT-116 were procured from ATCC (NCCS, Pune, India) for the calculated cytotoxic study. Milli-Q water was used in all experiments.

### 2.2. Preparation of Extract

*E. globulus* leaves were washed thrice with Millipore water and dried at ambient temperature in a dust-free chamber without sunlight exposure until constant weight was obtained. Ten grams of dried powder of the leaves mixed with 100 mL Milli-Q water was heated at exposure to 80 °C for 2 h, and the resulting light yellow solution was filtered, centrifuged and stored at 4 °C for further studies.

### 2.3. Green Synthesis of CeO_2_ NPs

Required amount of cerous nitrate hexahydrate (0.1N) was dissolved in 100 mL water, and then 100 mL aqueous plant extract was added dropwise through a peristaltic pump with stirring over 2–3 h. The pH of the mixture (initial pH 6.8) was adjusted to 8.2 by the addition of NaOH. After 24 h, pH of the mixture increased to 7.5 due to the formation of cerium hydroxide in the solution. The brownish yellow colored solution formed was centrifuged at 10,000 rpm for 15 min followed by ethanolic wash to remove impurities from solution. The obtained brownish yellow colored residue was dried at 80 °C for 2 h in a hot air oven and the dried powder was properly pulverized and then annealed at 400 °C for 3 h to obtain crystalline particles.

**Figure 1:**
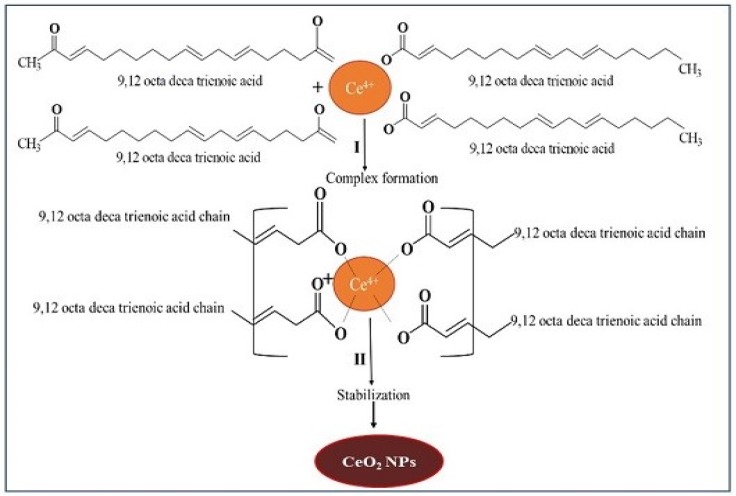
Mechanism of green synthesis of CeO_2_ NPs by using *E. globulus* leaf aqueous extract.

CeO_2_ NPs were prepared in two steps. In the first step, cerium nitrate was mixed with *E. globulus* leaf extract, which formed a three-dimensional bridge complex due to interaction of phytochemicals with cerium ions. In this step, *E. globulus* extract containing 9, 12 octadeca trienoic acid chains reacted with tetravalent Ce^+4^ via hydroxyl groups from two different chains to form network complex bridge with Ce^+4^ which conjugates to all the compounds present in the extract caused synergistic effect resulting in a complex structure. In the second step, the polymeric network chains suffered slow decomposition during calcination. The mechanism of CeO_2_ NPs formation is shown schematically in Figure 1.

### 2.4. Characterization Techniques

Powder X-ray diffractometer (Bruker D8 Advance X-ray Diffractometer) confirmed the crystalline texture of CeO_2_ NPs powder after CuKα radiation (λ = 1.54 Å). Crystallite size of CeO_2_ NPs was calculated by Scherrer’s formula as D = Kλ/(β cosθ), where λ = wavelength of X-ray radiation source (1.5406 Å), K = Scherrer constant as 0.9 for spherical particles, β = the width of the XRD peak at half height. 

Surface morphology was checked by FE-SEM and elemental composition was monitored by energy dispersive X-ray spectroscopy (EDAX). In addition, XRD was used to check phase purity which was cross-checked with selected area electron diffraction (SAED) pattern. SAED patterns were recorded using high resolution TEM (HR-TEM) equipped with Gatan CCD camera. DLS experiment identified particle size distribution of CeO_2_ NPs which was compared with XRD and HR-TEM results. Zeta potential was measured to predict stability using Horiba Scientific Nano Particii (SZ-100). CeO_2_ NPs dispersion was prepared by sonicating 1 mg NPs in 100 mL water at pH 7.5 for DLS and zeta potential measurement. Thermal analysis of the synthesized CeO_2_ NPs was carried out to determine weight loss with temperature. ATR-FTIR (Jasco-4100) at wave number range of 4000–400 cm^−1^ was used to trace functional groups attached to NPs as well as the extracts. UV-Vis-DRS study measured absorption intensity and band gap of the NPs in the wavelength ranges of 200–800 nm. The band gap of CeO_2_ NPs was calculated by using the following equation as $\alpha =c{\left(h\nu -E_{bulk}\right)}^{1/2}/hv$, where α is absorption coefficient, c is constant, hν is the photon energy and E_bulk_ is bulk ‘band gap’.

### 2.5. Photocatalytic Activity

The synthesized photocatalyst CeO_2_ NPs was used to degrade SY dye from aqueous solution using photoreactor (Heber multi-lamp photo reactor HML MP 88, mercury lamp of 8 W, 0.170 Å, voltage of 220–230 V at 50 Hz) under UV irradiation (λ 254 nm). The photodegradation efficiency (%) was assessed using 50 mL of SY dye aqueous solution (10 mg·L^−1^) mixed with varying amounts of photocatalyst (5, 10, 15, 20, 25 and 30 mg) by UV-Visible spectrophotometer at different time intervals.

### 2.6. Cytotoxicity Study of CeO_2_ NPs

#### 2.6.1. Cell Culture

Cell culture study was carried out using conventional method where Dulbecco’s modified Eagle’s medium (DMEM) containing 100 µg penicillin/mL, 100 U streptomycin/mL) and 10% heat-inactivated FBS was used in cell culture T25 flasks. Human A549 and HCT116 cell lines were cultured at 37 °C and 5% CO_2_ one day prior to the exposure studies with CeO_2_ NPs.

#### 2.6.2. Measurement of Cytotoxicity by MTT Assay

Cell viability was determined using MTT assay which is based on the conversion of the tetrazolium salt [3-(4,5-dimethylthiazol-2-yl)-5-(3-carboxymethoxyphenyl)-2-(4-sulfophenyl)-2 tetrazolium] to formazan crystals by mitochondrial NAD(P)H-dependent *oxidoreductase* enzymes. Briefly, cells were seeded at a density of 1 × 10^4^ cells/well into a 96 well plate and incubated overnight at 37 °C with 5% CO_2._ Then the cells were washed with PBS and media was discarded. The cells were then exposed to test chemical for 24 h at 37 °C with 5% CO_2_ and RH more than 80%. After removal of medium, 100 µL PBS was added to each well followed by addition of 100 µL MTT solution (0.5 mg/mL MTT in DMEM without FBS) and incubated for 1 h at 37 °C and 5% CO_2_. The formed formazan crystals were dissolved in 10% SDS (sodium dodecyl sulfate in 0.01 N HCl). MTT solution was then removed and 100 µL DMSO was added to each well and absorbance was measured using ELISA reader at 570 nm (EL800, Bio-Tech Instruments, Inc., Winooski, VT, USA). Additionally, one blank was used without CeO_2_ NPs for comparison.

Cell viability calculation was used to check the cytotoxic effect of the toxicants. MTT assay was performed in 96 well plate for negative control (only cell culture medium and cells were added), positive control (having cells and standard drug cisplatin, CDDP), experimental wells (having cells with CeO NPs), and blank samples (having only medium). All the experiments were carried out in triplicate. Cell viability was calculated as the ratio of the mean absorbance of replicated wells compared to that of the negative control wells. The experimental wells were compared to negative control with 100% growth. The IC_50_ values were obtained from the graph plotted using concentrations of CeO_2_ NPs along X-axis vs. cell viability (%) along Y-axis.

## 3. Results and Discussion

### 3.1. UV-Visible Spectroscopy

**Figure 2:**
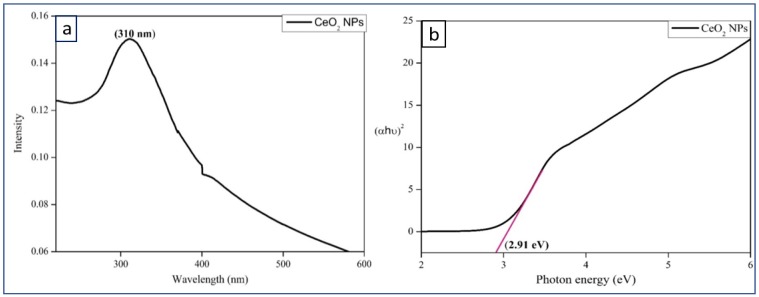
(**a**) UV-Visible spectrum of CeO_2_ NPs, (**b**) UV-DRS spectrum of CeO_2_ NPs.

Figure 2a shows the UV-Visible spectrum of CeO_2_ NPs dispersion which indicates the absorbance peak at 310 nm. UV-DRS (diffuse reflectance spectroscopy) spectrum of CeO_2_ NPs shows bandgap value as 2.91 eV. 

The optical band gap (E_g_) is found by the extrapolation of a Kubelka-Munk function fitted in a Tauc plot along the X-axis, as shown in Figure 2b. The band gap energy value indicates the semiconducting nature of the synthesized CeO_2_ NPs. 

### 3.2. Scanning Electron Microscopy (SEM) Analysis

**Figure 3:**
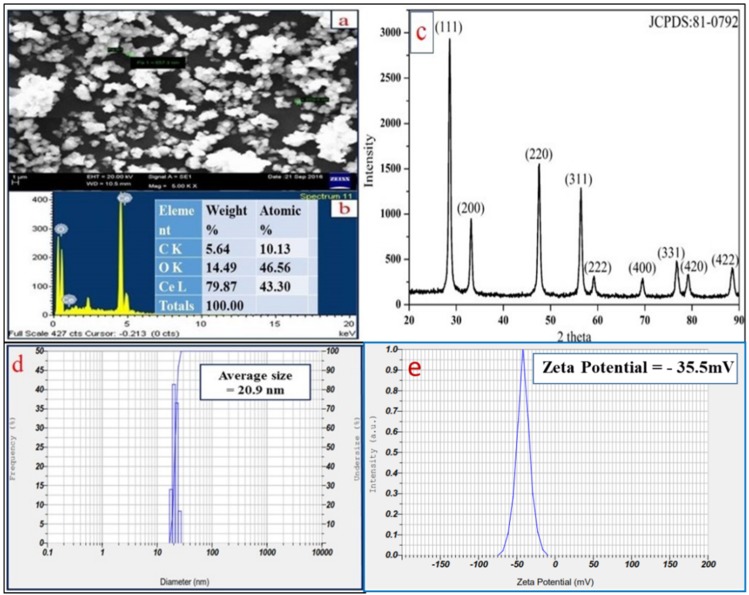
(**a**) SEM image of CeO_2_ NPs, (**b**) EDAX spectrum, (**c**) XRD diffractogram of synthesized CeO_2_ NPs, (**d**) particle size distribution, and (**e**) Zeta potential of CeO_2_ NPs.

Figure 3a represents the SEM image which shows spherical morphology of the particles. Figure 3b reveals the EDAX spectrum of CeO_2_ NPs with elemental analysis confirming the presence of “Ce” and “O” atoms with atomic percentage (79.87% and 14.49%) and weight percentage (43.30% and 46.56%), respectively. A very less intense carbon peak is seen due to the contribution of carbon tape attached on the stub surface.

### 3.3. Powder X-ray Diffraction Analysis

Figure 3c shows XRD pattern of the synthesized CeO_2_ NPs exhibiting the peaks at 2θ values with hkl indexing of 28.54° (111), 33.07° (200), 47.47° (220), 56.33° (311), 59.07° (222), 69.40° (400), 76.68° (331), 79.06° (420), and 88.41° (422). This pattern indicates crystal system of cubic phase structure having space group number Fm3m (225), unit cell volume (158.55 Å) and lattice parameters (a = b = c = 5.4124 Å) [20]. The crystallite size of CeO_2_ NPs is 20.72 nm as per calculation by the Scherrer’s formula [21].

### 3.4. Dynamic light Scattering (DLS) Analysis

The stability of CeO_2_ NPs is stated in terms of zeta potential. It is defined as the potential difference around surface charge groups associated on the NPs surfaces, and dispersed solvent medium containing groups of opposite charge. Basically, higher negative value of zeta potential indicates more repulsion between the particles which minimizes aggregation and results in higher stability of bio-fabricated NPs [22]. Figure 3d shows the particle size distribution with an average size of CeO_2_ NPs as 20.9 nm. Figure 3e describes the measured zeta potential value of CeO_2_ NPs as −35.5 mV at pH 7.5, which indicates higher dispersibility of the NPs with high stability.

### 3.5. Transmission Electron Microscopy (TEM) Analysis

**Figure 4:**
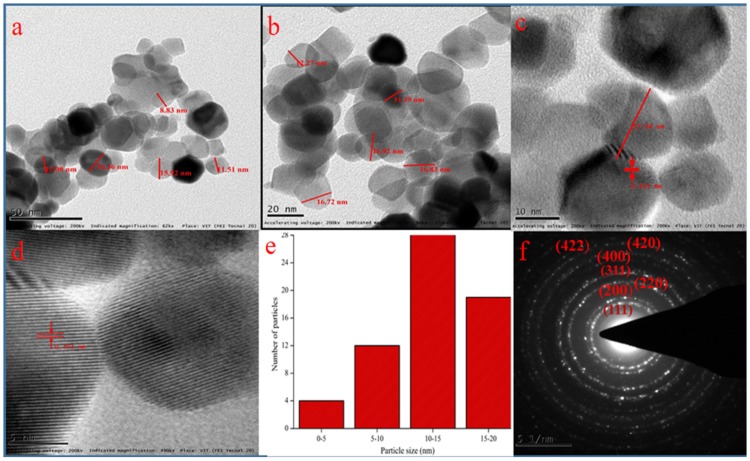
TEM images of CeO_2_ NPs at different magnifications: (**a**) 50 nm, (**b**) 20 nm, (**c**) 10 nm, (**d**) 5 nm; (**e**) particle size histogram, (**f**) SAED pattern with hkl values.

Figure 4a–c shows the transmission electron micrographs representing the particles sizes of 15–20 nm. Figure 4d specifies HRTEM micrograph which confirms the formation of CeO_2_ particles having fringe space of 3.1 Å The results are in close agreement with XRD data with (111) plane (3.24 Å) of CeO_2_ NPs and the low crystalline nature of particles resembles its high reactivity to radicals [23]. Figure 4f represents the SAED pattern and hkl values which supports the XRD pattern of polycrystalline particles. 

### 3.6. Fourier-Transform Infrared Spectroscopy (FT-IR) Analysis

**Figure 5:**
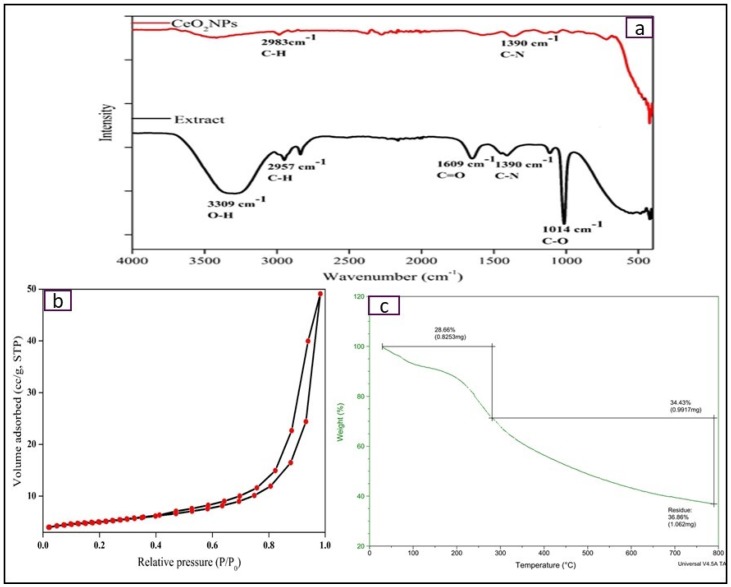
(**a**) Nitrogen adsorption–desorption isotherm of CeO_2_ NPs; (**b**,**c**) TGA thermogram of CeO_2_ NPs.

FTIR analysis is carried out to find out the functional groups attached to NPs surface as well as in phytochemicals present in *E. globulus* extract (Figure 5a). FTIR spectra show the presence of phenolic OH group (absorption band at 3350 cm^−1^), and carboxyl (C=O) group (absorption band at 1690 cm^−1^) which reduces Ce^4+^ ions into CeO_2_ NPs [24]. Other absorption bands such as 2954 and 2833 cm^−1^ correspond to the stretching vibrations of alkanes, 1390 cm^−1^ for the CH_2_ bending, 1110.8 cm^−1^ for C–O bending vibrations. Additionally, lower shift functional groups correspond to 940.3 cm^−1^ for C–O and 640.3 cm^−1^ for Ce–O bond respectively. It resembles the formation of CeO_2_ NPs and the polyphenol groups present in the *E. globulus* extract behave both as reducing agent to cerium ions and stabilizing agent of CeO_2_ NPs.

### 3.7. Surface Area and Porosity Analysis

Specific surface area and pore size distribution of the synthesized CeO_2_ NPs are calculated using nitrogen adsorption–desorption isotherms. Figure 5b shows the nitrogen adsorption–desorption isotherm at 77 K and the measured Brunauer–Emmett–Teller (BET) specific surface area of CeO_2_ NPs is 40.96 m^2^/g, which is much higher than commercial CeO_2_ NPs (8.50 m^2^/g) [25]. Additionally, Barrett–Joyner–Halenda (BJH) pore size distribution curve displays a narrow pore size distribution with a calculated pore diameter of 7.8 nm. These isotherms are considered as Type IV isotherms [26].

### 3.8. Thermogravimetric Analysis (TGA)

Thermogravimetric analysis shows weight loss of the compound with respect to temperature. Figure 5c displays the TGA thermogram of the bio-synthesized CeO_2_ NPs against temperature (@ 5 °C/min) under nitrogen gas atmosphere, with two-step weight losses within the temp range of 0–800 °C. The first step is associated with 11.6% weight loss in the temperature range of 20–200 °C due to elimination of water molecules and absorbed moisture. Second major weight loss of 18.6% occurred in 300–400 °C due to oxidative decarboxylation of Ce(NO_3_)_3_ to CeO_2_ [4].

### 3.9. Photocatalytic Degradation of SY Dye

**Figure 6:**
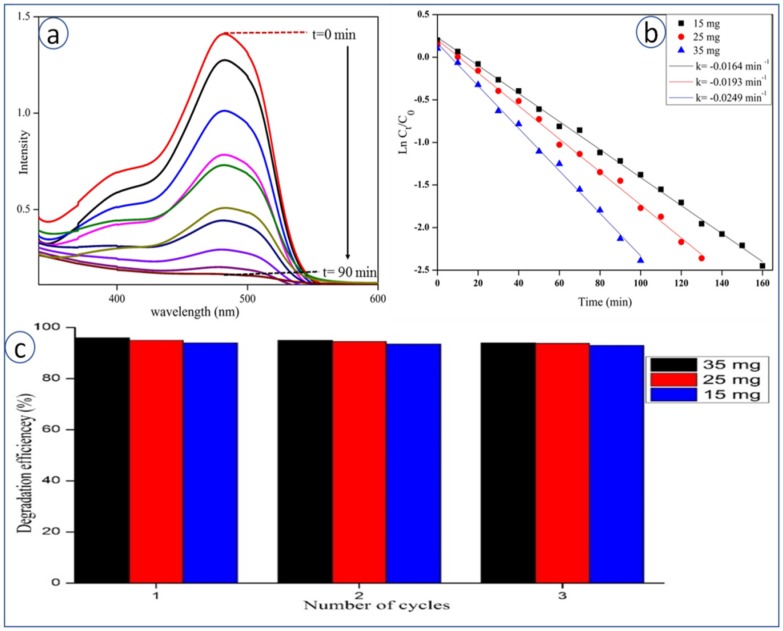
(**a**) UV-Visible absorbance spectra of SY dye during degradation with CeO_2_ NPs, (**b**) kinetic studies of SY dye degradation with CeO_2_ NPs, (**c**) recyclability study of different amounts of catalysts recovered towards degradation of SY Dye.

The photocatalytic activity of the synthesized CeO_2_ NPs was studied during the degradation of SY dye under UV-irradiation. It was observed that the intensity of SY dye colour did not change in the dark, during a time period of 1–2 h, with or without CeO_2_ NPs. SY dye was found to degrade only under UV-irradiation, which was confirmed by UV-Visible spectroscopy (no appearance of dye peak) (Figure 6). Figure 6a describes the UV-Visible spectra for degradation kinetics of SY at different time intervals (from 0 to 90 min) at λ_max_ 520 nm. It clearly shows that the colour intensity of SY decreases with time in the presence of CeO_2_ NPs. Initially, SY was found to degrade up to 90.7% after 90 min of UV irradiation. Figure 6b represents the plot of ln (C_t_/C_0_) versus irradiation time (min), and a linear relationship is obtained based on the following equation as Ln(C/C_0_) = −kt, where C_0_ is the concentration of SY at time t “0 min”, C is the concentration of SY at time “t min” and k is the slope constant. Slope value obtained for SY with optimized catalyst dosage (35 mg) is k = 0.0250 min^−1^, which follows pseudo first-order kinetic rate. The recyclability of the catalyst is worked out and up to three cycles, whereby no significant change in percentage of degradation is seen, as shown in Figure 6c.

### 3.10. Cytotoxicity Study

**Figure 7:**
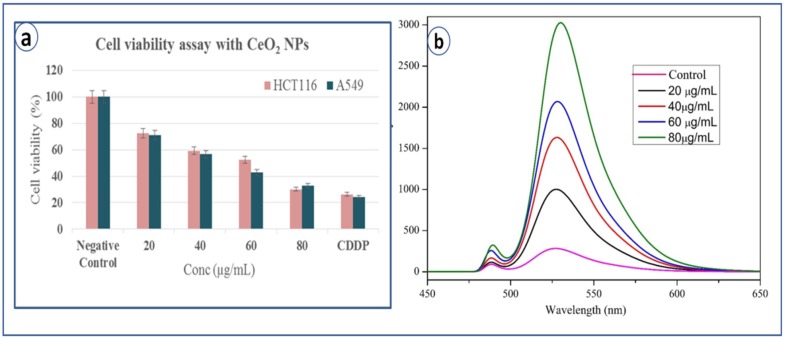
(**a**) Cell viability test by MTT assay with CeO_2_ NPs, (**b**) ROS emission spectra of control and CeO_2_ NPs.

Cytotoxicity study of CeO_2_ NPs is performed against A549 and HCT 116 cancer cell lines. The cell viability is analyzed by MTT assay and IC_50_ (Half maximal inhibitory concentration) value obtained is 45.5 µg/L for A549 cell line and 58.2 µg/L for HCT 116, which clearly suggests that the biosynthesized CeO_2_ NPs are more toxic to A549 cell lines compared to HCT 116 cell lines. It can be noted that it is toxic to both the cell lines suggesting its potential as anticancer agent. The cytotoxicity of CeO_2_ NPs is compared with standard drug cisplatin (CDDP), which is mostly similar at high dose of CeO_2_ NPs (80 µg/mL) (Figure 7a).

### 3.11. Estimation of Reactive Oxygen Species (ROS) Generation by DCF Method

**Figure 8:**
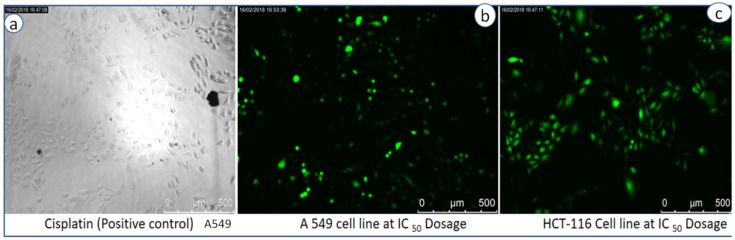
ROS fluorescence microscopy images of (**a**) cisplatin (positive control), (**b**) A549 cell lines, and (**c**) HCT 116 cell lines at IC_50_ dosages.

To estimate intracellular ROS generation, a cell-permeable non-fluorescent dye 2, 7 dichlorofluorescein diacetate (DCFH2-DA) is used, which can be de-esterified intracellularly to highly fluorescent 2-dichloro fluorescein (DCF+) upon oxidation [27]. It is commonly observed that metal oxide NPs interact with cell membranes and proteins inside the cells and release ROS species leading to the imbalance in redox state of cell, which causes oxidative stress and cell death [17]. Pro-oxidant functional clusters present on the reactive surface of NPs play a major role in kinetics of toxicity, and disrupts the cell signaling and immune defense mechanism [18]. Recently, our research group has reported the death of cancer cell lines by SnO_2_ NPs due to dose-dependent production of ROS species [19]. Basically, reactive oxygen species (i.e., free radicals) generated in cells under stress conditions interact with cytoplasm, proteins and mitochondria resulting in cell destruction [28]. Figure 7b explains the comparative fluorescence emission spectra of both control and CeO_2_ NPs at different concentrations (20, 40, 60, 80 µg/mL) at an emission wavelength of 523 nm. It clearly exhibits ROS generation by CeO_2_ NPs with increasing intensities of DCF dye, which is directly proportional to the dose. Compared to the control, bio-synthesized CeO_2_ NPs show higher emission intensity due to more generation of ROS species or more cell deaths. ROS is generated from reduced nicotinamide adenine dinucleotide dehydrogenase II in the respiratory chain by the auto-oxidation process [28,29,30,31,32]. In addition to the emission spectra of ROS, fluorescence microscopy images are captured for negative control and exposure of IC_50_ concentration of CeO_2_ NPs dispersion shows the ROS emission with green colour spotted images, which indicate both cell lines generated ROS in the presence of CeO_2_ NPs (Figure 8a,b).

## 4. Conclusions

In summary, CeO_2_ NPs were synthesized using *E. globulus* leaves extract via a green method. Synthesized NPs show photoluminescence, cubic structure, and spherical shape with particle size ranging from 8–20 nm, which are confirmed by various analytical techniques like XRD, SEM and TEM. The bio-synthesized CeO_2_ NPs are a superior photocatalyst compared to other photocatalysts due to oxygen vacancies, decreased band gap, surface defects and lower recombination tendency of the electron–hole pair. The synthesized nanoparticles remain stable up to four recycles without change in photocatalytic efficiency. Additionally, CeO_2_ NPs are cytotoxic to human lung A549 and colon HCT 116 carcinoma cell lines, with IC_50_ values of 45.5 and 58.2 µg/L respectively. The current results suggest the promising potential of CeO_2_ NPs for potential anticancer therapy.
